# Arterial Thrombosis Following Umbilical Artery Catheterization: Visualising Uncommon Neonatal Intensive Care Unit (NICU) Complications

**DOI:** 10.1002/ajum.70023

**Published:** 2025-09-25

**Authors:** Andrea Calandrino, Alessia Pepe, Francesco Vinci, Luca Antonio Ramenghi

**Affiliations:** ^1^ Neonatal Intensive Care Unit, Department of Maternal and Neonatal Health IRCCS Istituto Giannina Gaslini Genoa Italy; ^2^ Department of Neuroscience, Rehabilitation, Ophthalmology, Genetics, Mother and Child Health, School of Medical and Pharmaceuticals University of Genoa Genoa Italy

**Keywords:** heparin, internal iliac artery, NICU, skin lesion, thrombosis, UAC

## Abstract

**Background:**

Right internal iliac artery (RIIA) thrombosis is an extremely rare but serious complication associated with umbilical artery catheter (UAC) malposition in neonates in the Neonatal Intensive Care Unit (NICU). Timely diagnosis and appropriate management are essential to prevent long‐term sequelae.

**Case Report:**

We present the case of a term neonate with hypoxic–ischaemic encephalopathy (HIE), who developed RIIA thrombosis secondary to UAC malposition, highlighting the role of bedside Doppler ultrasound in diagnosis and monitoring, and the success of a conservative therapeutic approach.

**Discussion:**

A term male neonate with HIE was undergoing therapeutic hypothermia when violaceous macular skin lesions appeared on the right buttock and loin shortly after UAC insertion. Imaging confirmed malposition of the catheter in the RIIA. The catheter was promptly removed, and the patient was closely monitored both clinically and with serial Doppler ultrasounds. Approximately 6 h after catheter removal, Doppler ultrasound revealed a clot in the RIIA. The patient was managed conservatively with continuous infusion of unfractionated heparin (10 IU/kg/h) and topical anti‐inflammatory therapy. The skin lesions resolved within 36 h, and Doppler at 48 h post‐removal confirmed re‐established arterial flow. No invasive interventions were required. This case underscores the importance of careful catheter placement and the utility of bedside Doppler ultrasound in detecting and monitoring vascular complications. Moreover, prompt recognition and conservative treatment of arterial thrombosis can result in optimal outcomes, even in neonates with complex clinical conditions such as birth asphyxia.

**Summary**

- This case report describes a newborn who developed a skin lesion after a common procedure called umbilical artery catheterisation.
- The lesion was caused by a clot due to a misplaced catheter.
- A Doppler ultrasound played a crucial role in promptly identifying the clot, ruling out other possible causes, and guiding timely treatment with blood thinners and topical therapy.
- Thanks to early ultrasound assessment, the baby recovered quickly without the need for invasive procedures.
- This case highlights the essential role of ultrasound in detecting and managing vascular complications in newborns, ensuring safer outcomes in neonatal intensive care.

Umbilical artery catheterisation (UAC) is a commonly employed procedure for the management of critically ill neonates requiring continuous haemodynamic monitoring. While generally considered safe, UAC carries the risk of various complications, including malposition, bloodstream infections, thrombosis, tip migration, and extravasation. These complications can significantly increase morbidity and mortality [1].

The reported case pertains to a male term neonate diagnosed with hypoxic–ischemic encephalopathy and a candidate for therapeutic hypothermia (TH) who developed violaceous macular skin lesions on the right buttock and loin in the first hours following UAC (Figure 1, panels A, B). The X‐ray immediately after the procedure showed catheter mispositioning in the right internal iliac artery (RIIA) (Figure 1, panel C), which was immediately removed.

**FIGURE 1:**
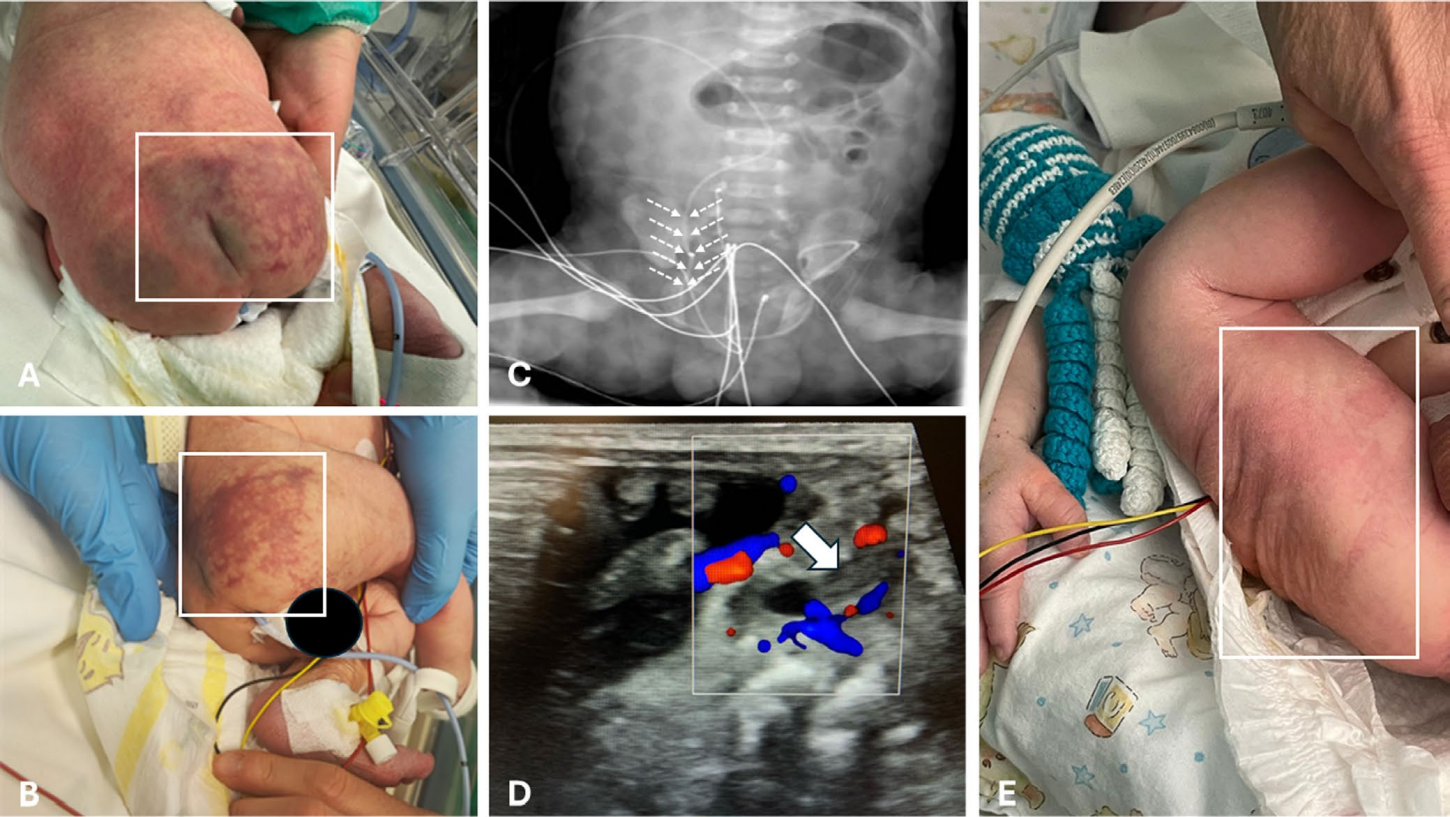
Violaceous macular skin lesions on the right buttock and loin, panels A and B (white boxes). X‐ray showing misplacement of the UAC in the right internal iliac artery, panel C (white dashed arrows). Doppler ultrasound of the right internal iliac artery showing a clot in the vessel lumen and absence of blood flow, panel D (white arrow). Resolution of the cutaneous lesions with near complete restitution to baseline after 48 h of treatment with unfractionated heparin and topical therapy, panel E (white box).

These lesions, initially suspected to be subcutaneous fat necrosis related to TH, appeared approximately 6 h after the removal without any associated symptom; a Doppler ultrasound was conducted, which revealed the presence of a clot in the RIIA (Figure 1, panel D). Consequently, an infusion of unfractionated heparin (10 IU/kg/h) and topical anti‐inflammatory therapy was promptly administered.

The skin lesion completely resolved within 36 h of treatment (Figure 1, panel E), and a follow‐up Doppler ultrasound at 48 h demonstrated restored circulation in the artery.

Adverse events (AEs) resulting from central line misplacement in neonates, while uncommon, can be severe. Umbilical venous catheters (UVCs) have an AE rate of 13.4%, while UACs are associated with an AE rate of 9%. Among these, vascular thrombosis is reported in approximately 1% to 3% of UAC cases [1].

In this case, a clot in the RIIA led to ischemic involvement of the skin, presenting as a violaceous macular lesion. The differential diagnosis included subcutaneous fat necrosis due to TH [2], as well as other conditions such as vasculitis, infection, and congenital anomalies. The Doppler ultrasound was instrumental in ruling out these alternatives [3].

The management of arterial thrombosis in critically ill newborns, particularly those with birth asphyxia, who suffer from concomitant impaired liver function resulting in a disrupted coagulation balance, thus making coagulation management particularly challenging [4]. This case highlights the importance of vigilant monitoring for vascular complications following UAC placement, as well as the effectiveness of rapid, conservative management in preventing the need for more invasive interventions.

## Author Contributions

**Andrea Calandrino:** conceptualization (lead), data curation (lead), formal analysis (equal), funding acquisition (equal), investigation (equal), methodology (lead), project administration (lead), resources (equal), software (lead), supervision (supporting), validation (equal), visualization (lead), writing – original draft (lead), writing – review and editing (supporting). **Alessia Pepe:** conceptualization (supporting), data curation (equal), formal analysis (equal), funding acquisition (supporting), investigation (equal), methodology (supporting), project administration (supporting), resources (supporting), software (supporting), supervision (supporting), validation (equal), visualization (supporting), writing – original draft (equal), writing – review and editing (supporting). **Francesco Vinci:** conceptualization (supporting), data curation (supporting), formal analysis (supporting), funding acquisition (supporting), investigation (equal), methodology (equal), project administration (supporting), resources (supporting), software (supporting), supervision (supporting), validation (equal), visualization (lead), writing – original draft (equal), writing – review and editing (supporting). **Luca Antonio Ramenghi:** conceptualization (equal), data curation (supporting), funding acquisition (lead), methodology (equal), project administration (lead), resources (lead), supervision (lead), validation (supporting), writing – review and editing (equal).

## Ethics Statement

The study was conducted in compliance with the terms of the Helsinki Declaration and written informed consent for the enrolment and for the publication of individual clinical details was obtained from parents. In our country, namely Italy, this type of clinical study does not require Institutional Review Board/Institutional Ethics Committee approval to publish the results.

## Consent

Written informed consent for publication both in online and printed versions of clinical details and/or clinical images was obtained from the parents of the patient. A copy of the consent form is available for review by the Editor of this journal.

## Conflicts of Interest

The authors declare no conflicts of interest.

## Data Availability

All clinical data reported in this manuscript are available for review by the Editor of this journal upon reasonable request. These data have not been previously shared or published elsewhere.
